# Role of Macromolecular Crowding on the Intracellular Diffusion of DNA Binding Proteins

**DOI:** 10.1038/s41598-017-18933-3

**Published:** 2018-01-16

**Authors:** Pinki Dey, Arnab Bhattacherjee

**Affiliations:** School of Computational and Integrative Sciences, Jawaharlal Nehru University, New Delhi, 110067 India

## Abstract

Recent experiments suggest that cellular crowding facilitates the target search dynamics of proteins on DNA, the mechanism of which is not yet known. By using large scale computer simulations, we show that two competing factors, namely the width of the depletion layer that separates the crowder cloud from the DNA molecule and the degree of protein-crowder crosstalk, act in harmony to affect the target search dynamics of proteins. The impacts vary from nonspecific to specific target search regime. During a nonspecific search, dynamics of a protein is only minimally affected, whereas, a significantly different behaviour is observed when the protein starts forming a specific protein-DNA complex. We also find that the severity of impacts largely depends upon physiological crowder concentration and deviation from it leads to attenuation in the binding kinetics. Based on extensive kinetic study and binding energy landscape analysis, we further present a comprehensive molecular description of the search process that allows us to interpret the experimental findings.

## Introduction

Crucial cellular processes such as transcription, DNA replication, DNA damage repair, etc. rely on recognition of the target sites located on DNA followed by formation of specific complexes by a class of proteins, often referred as DNA binding proteins (DBPs). The seminal *in vitro* experiment by Riggs *et al*.^1^ was the first ever to explore the binding of one of such DBPs, namely the lac repressor, which was found to locate its operator region with an incredible speed and efficiency. The finding elicited a series of studies using analytical^2–5^ and computational approaches^6–9^ that probed the target search mechanism of DBPs at various levels of complexity. It was proposed that protein expedites its search process by lowering the dimension of the search space, where it performs 1D diffusion along the DNA contour in addition to 3D diffusion^10^. The 1D diffusion is further classified into two types: the sliding motion through the DNA major grooves and the small jumps performed by the protein on DNA surface (hopping). Each of these modes of translocation is confirmed experimentally with the recent advances in spectroscopic techniques^11–14^ and single molecule experiments^15,16^. Despite the knowledge of mechanistic details and other molecular determinants that might affect the target search, the complete understanding of the process is yet to be achieved. The major challenge is posed by the cellular environment, which unlike to the *in vitro* condition is densely crowded (≈10–40% w/v) by the presence of other proteins and macromolecules. Apparently, such a dense media is supposed to hinder the diffusion of a protein searching for its binding site on the genomic DNA. Recent experiments, however, suggest the opposite and the crowders are indeed found to facilitate the target search process of proteins on DNA and enhance their enzymatic activity^17,18^. Evidently, the action of macromolecular crowders can not be described merely through the excluded volume interactions, rather factors originating from a high concentration of inert crowders should also be taken into account^19^. For example, colloidal physics arguments suggest that crowding causes depletion between the constituents of a system^20^, which is essential for its thermodynamics^21^. Also, the inclusion of crowders enhances the viscosity of the bulk solution (macroscopic viscosity) but at the same time, their unavailability inside the depletion layer results in a constant microscopic viscosity. Consequently, a protein searching for its target site experiences two distinctly different viscous media when performing 3D diffusion in bulk and 1D diffusion along the DNA contour respectively. How such duality in the cellular environment affects the target search dynamics? The issue has gained much attention lately^22–25^ but is far from being solved. The ambiguity arises as in some cases target search time remains unchanged even in the presence of crowders^26^, whereas some reports confirmed that crowding agents could promote ‘facilitated diffusion’^17^. Unclarity also persists in the mechanism of facilitation in target search process. A recent study based on molecular simulations has proposed that nonspecific interaction between a searching protein and the crowder molecules promotes hopping, which is a faster mode of translocation compared to the sliding and thereby reduces the target search time^27^. This is, however, in contrast to the observation reported by Luo *et al*.^28,29^ that crowding agents prevent the escape of the searching protein from the DNA surface and thus increases the sliding propensity and the average distance covered per sliding events. However, how the sliding being a slower mode of diffusion can boost the target search kinetics remains unaddressed. The above arguments necessitate an in-depth study to elucidate the molecular description of how the cellular milieu, replete with macromolecular crowders, affect the target search dynamics of DBPs and provide a theoretical foundation in order to unify the current observations. For this, we perform large scale computer simulations using an appropriately chosen coarse-grained model of protein and DNA with spherical crowders and systematically analyse every facet of the target search mechanism. We show that the crowder molecules restrain the DNA by forming a depletion layer around it, the width of which decreases with increasing crowder concentration. On the other hand, the searching protein starts communicating more and more with the crowders as the concentration of the latter rises. The intriguing combination of these two competing factors modulates the mode and the efficiency of the target search dynamics. Furthermore, the kinetic experiments and the analysis of binding energy landscape reveal the molecular origin of the facilitated diffusion of DBPs in the presence of macromolecular crowders. Our result also underscores that the fastest kinetics is achieved only at physiological crowder concentration, a deviation from which results in attenuation of the binding kinetics.

## Results

**Figure 1:**
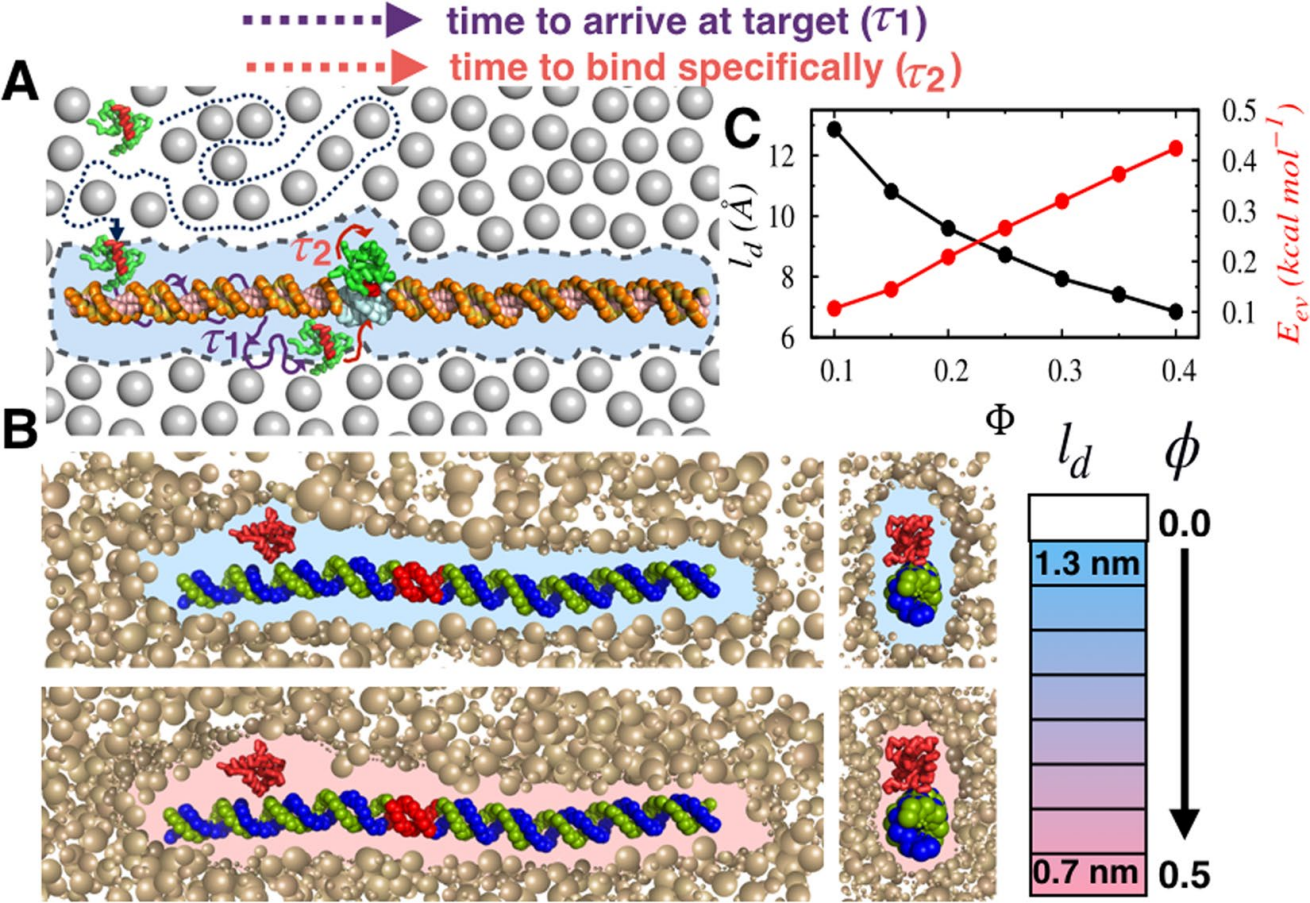
Protein searches for its target DNA sequences inside crowded cellular milieu. (**A**) Schematic representation of the target search process. The protein performs 3D diffusion in the bulk solution until it senses the DNA. The time required by the protein to reach the target site after being associated nonspecifically with the DNA includes the time taken by the protein to scan the DNA non-specifically, denoted by τ_1_ and the time required to orient and bind specifically to the DNA, denoted by τ_2_. (**B**) Schematic representation of formation of the crowder-DNA depletion layer originated due to the dynamics of these molecules. With increasing volume fraction of crowders (Φ), the depletion region narrows down. The position of crowders are completely random and their sizes are identical, although some of them appear smaller due to their spatial distribution. (**C**) The variations of the width of the depletion region, *l*_*d*_ ($\mathring{\rm A}$) and the excluded volume energy, arising out of crosstalk between the crowder and the searching protein are presented with respect to Φ.

To probe the role of macromolecular crowders, we dissect the simulation generated target search trajectories of Sap-1 into two distinct phases. As shown in Fig. 1, (i) the process initiates when the searching protein from the bulk comes close to the DNA, attaches nonspecifically to it and diffuses along the DNA contour in the quest of target DNA site. The process lasts until the protein recognizes the target DNA site in τ_1_ time through a combination of 1D and 3D modes of diffusion. (ii) After reaching the target site, the protein takes τ_2_ time to reorient appropriately and bind specifically by establishing all the specific protein-DNA contacts. The previous study has suggested that the orientation time is strongly dependent on the sequence composition of the searching protein^30^. We, however, are interested in probing the role of crowders in regulating this and the previous phase of the target site finding of the searching protein. To begin with, we measure the average distances (*l*_*d*_) between each DNA base pair and the crowder molecule nearest to it. Therefore, *l*_*d*_ approximately measures the width of the depletion region that separates the crowder cloud from the DNA surface. To this end, we emphasize that the depletion layer in our model has an entropic origin, and no artificial condition has been imposed. The dynamics of the crowder and the DNA molecules are such that they allow only restricted adjacency between the molecules, resulting in the formation of a depletion volume in between. It is further supported by the fact that the average of the shortest crowder-crowder distances at Φ = 0.4 (where Φ is the fraction of the total volume occupied by the crowders) is ~0.32 nm (see Fig. S1 for more details). The same between the crowder-DNA (*l*_*d*_) at similar crowder concentration is, however, much wider (~0.7 nm). Had it been due to the radii of the interacting residues, the crowder-crowder depletion region would have been wider than that of the crowder-phosphate (radius of phosphate bead in DNA is much smaller than that of crowder molecules) depletion region. This indicates the pivotal role played by the DNA dynamics. Here, we carefully modeled the DNA molecule adopting an implicit solvent approach as shown by Pablo *et al*. (see Methods for details) to precisely capture its dynamics in solution^31^. The estimation of depletion layer is different for polymeric crowder molecules and is typically done through calculating the radius of gyration (*R*_*g*_) of the polymer^17^. We, however, find an analogy between both the approaches. For example, in our model, *l*_*d*_ varies with crowder concentration (see Fig. 1B) and the result in Fig. 1C shows that *l*_*d*_ decreases from ≈1.28 nm to ≈0.68 nm with Φ value increasing from 0.1 to 0.4 (which is equivalent to 0.003–0.15% w/v). The reported *l*_*d*_ in experiments is ≈0.9 nm for a dilute solution (5% w/v) of PEG 600 crowders^17,32^. Although the crowder concentration in our model is of lower magnitude than that of the experimental condition, we argue that both fall under the dilute solution category. For semi-dilute to crowder-dense solutions, the depletion layer shares a complex relationship with crowder concentrations^17^. It is also interesting to note that the width of the depletion layer in the present model agrees well with the dimension of third (~1 nm) and second hydration shells (~0.7 nm) around a B-DNA^33,34^ molecule. The analogy indicates that a depletion volume forms under *in vivo* condition as well, although the driving force is ‘hydration interactions’ that arranges the adjacent water molecules to form a “spine of hydration”^35^. Formation of similar depletion region because of short-range repulsive interactions (‘depletion forces’) has been observed by a model study of colloid adsorption on lipid membranes^36^. Within this micro-diffusion limit viscosity remains unaltered even if the crowder concentration changes in the bulk. Previous studies have confirmed the importance of such hydration shells in determining the biology of DNA molecule and regulating its interactions with transcription factors and ligands^37–39^. The decrease in the width of depletion layer with increasing Φ is due to the fact that increased crowder concentration excludes available volume in the system, resulting in a narrower depletion region. This is also in line with the experimental observations that the polymer crowders overlap to form a noncovalent network with a certain average mesh size^17^ if their concentration exceeds a threshold concentration. The interacting proteins and DNA are embedded on the mesh, the size of which varies with radius of gyration (R_g_) of the polymeric crowder molecules. Notably, R_g_ decreases with the increasing crowder concentration and thereby lowers the width of the depletion layer around the DNA. In addition to width of the depletion layer, the rise in crowder concentration also affects the communication between crowders and the searching protein. The result in Fig. 1C implies that with increasing Φ, crowder molecules collide more frequently with the protein, leading to a hike in the excluded volume interactions (*E*_*ev*_) between these two. How the interplay of these two factors, namely *l*_*d*_ and *E*_*ev*_ regulates the target search mechanism of DBPs?

### Crowders affect nonspecific target search and specific complex formation differently

**Figure 2:**
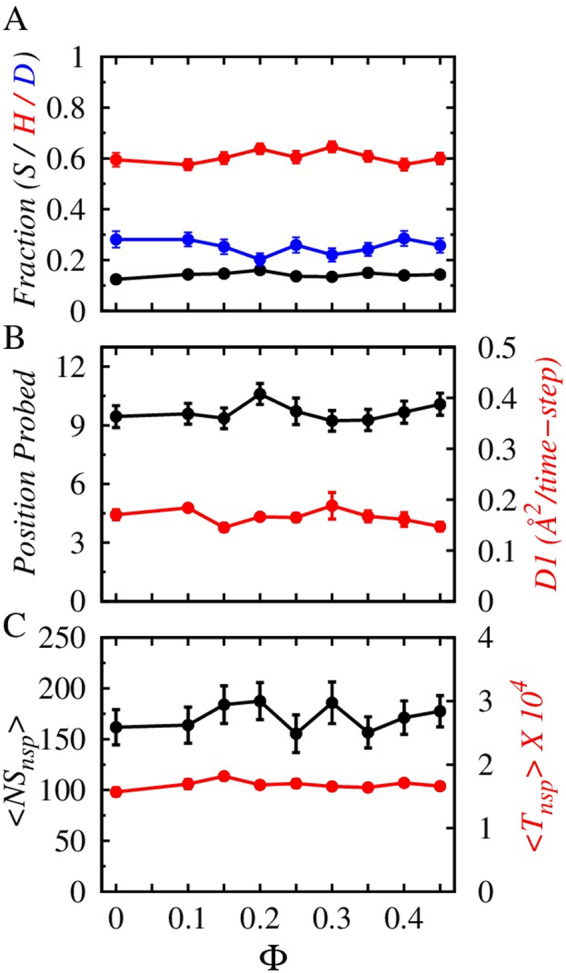
Nonspecific target search of protein as a function of crowder concentration, Φ. (**A**) Effect of increasing volume fraction of crowders (Φ) on non-specific search dynamics (sliding (S), hopping (H) and 3D diffusion (**D**)) at 140 mM salt concentration. (**B**) Variation in the Position-probed by the DBP (black line) and the 1D diffusion coefficient (D_1_, denoted by the red line) as functions of Φ. (**C**) The variation of an average number of non-specific sliding events (<NS_nsp_>, denoted by the black line) and the average duration (<T_nsp_>, indicated by the red line) of a sliding event is shown with respect to Φ.

Figure 2 presents an in-depth analysis of the effects of crowders on the nonspecific target search mechanism of the searching protein. It (Fig. 2A) shows that the propensities of different modes of translocation (sliding, hopping and 3D diffusion) remain roughly constant with the variation in crowder concentration. It is not only the mode of search, rather the protein exhibits a very robust one-dimensional diffusion ability along the DNA contour (D_1_) irrespective of the bulk crowder concentration (Φ), as can be seen in Fig. 2B. This is also supported by the observation that the average number of positions that are probed by the searching protein while diffusing nonspecifically along the DNA are very much similar (see Fig. 2B) for all Φ values. We further investigate the mechanistic details of the nonspecific search dynamics to exclude the possibility that a varying number of events of a particular search mode with varying durations per event may still yield a constant propensity for the search mode. In Fig. 2C, we present the average number and duration of sliding events as functions of Φ. We surprisingly note that both the number and the duration of sliding events remain invariant with the change in crowder concentrations. These clearly suggest that crowder has no or marginal impacts on the nonspecific target search mechanism of DBPs. This is in line with a previous site transfer assay experiment^17^, where the nonspecific binding of the protein to DNA was found to be independent of crowder concentration. The insight, however, comes from our study and can be explained by considering the following. For nonspecific search the protein has to enter inside the depletion regime, the width of which is ~12–7 Å. A protein positioned further away than this distance experiences weak electrostatic attraction from the DNA molecule. Within the depletion region, the protein dynamics is regulated by two competing effects of crowder molecules acting in harmony. As the crowder concentration increases the depletion region narrows down (Fig. 1C), thereby forces the protein to stay close to the DNA. The searching protein feels stronger electrostatic attraction from the DNA molecule (see Fig. S2) that typically enhances the sliding propensity of the protein. One can compare the situation with decreasing salt condition. As the salt condition decreases, the effective electrostatic attraction between the recognition region of the searching protein and DNA increases, resulting the former closely probe through the DNA major groove in sliding mode^6^. Our result in Fig. 2A however, does not reflect any increment in the sliding propensity. This is attributed towards the contrasting role of crowder molecules, where increasing crowder concentration increases the collision frequency with the protein (corresponding *E*_*ev*_ rises in Fig. 1C). The respective pushes from the crowder molecules obstruct the smooth sliding of the protein along the DNA major grove and compel it to perform short jumps on the DNA surface, which can promote hopping dynamics^25^. Since, the sliding is complementary to hopping dynamics (see Methods), the impacts of increasing crowder concentration to promote both of them simultaneously by lowering the width of depletion region and increasing the protein-crowder collisions outweigh each other. This results in the target search propensities, average number of events in each search mode and their durations independent of the crowder concentration during the nonspecific search regime. It should however, be noted that the role of crowder is entirely different in bulk, where the protein is far away from the DNA surface and performs 3D diffusion. Figure S3 indicates that the 3D diffusion coefficient decreases as the crowder concentration in bulk increases. Besides the impacts of crowder molecules, the protein inside the depletion region experiences a constant viscosity (microscopic viscosity), which is independent of crowder concentration unlike to that of the bulk solution. This explains the origin of robust diffusion ability (constant D_1_ values, see Fig. 2B) of the protein irrespective of bulk crowder concentrations.

**Figure 3:**
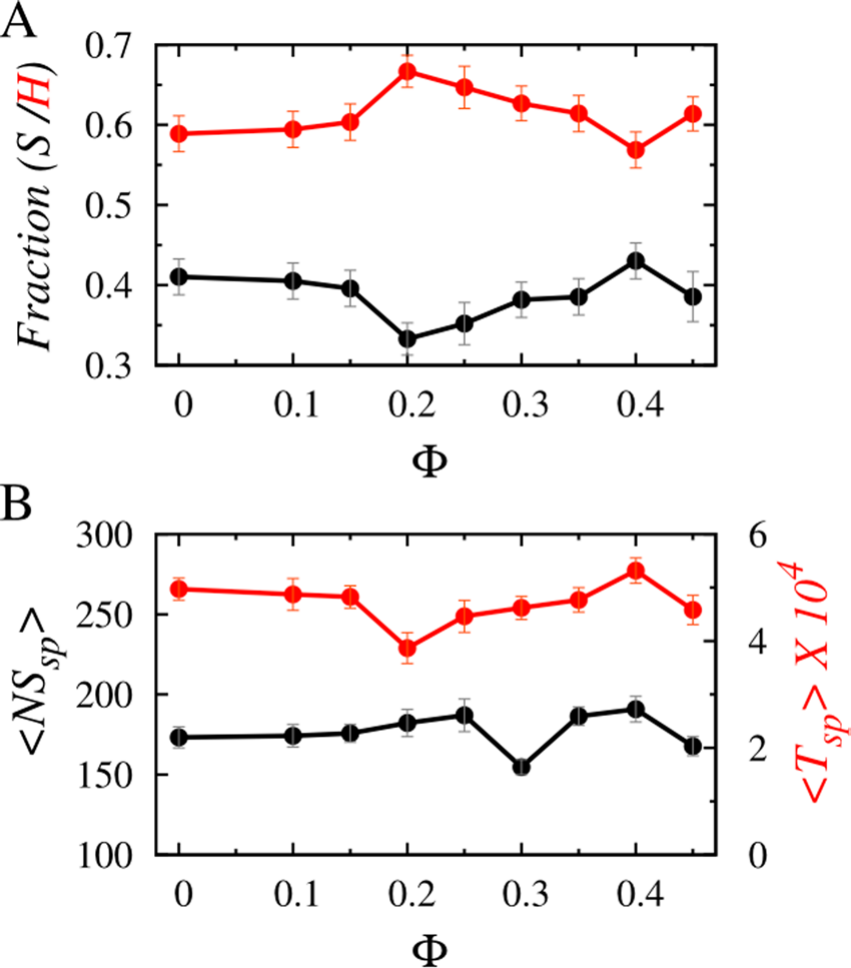
Impacts of macromolecular crowders on the specific protein-DNA complex formation. (**A**) Effect of increasing volume fraction, Φ on the specific target search dynamics, (S) and (H) of the interacting protein at 140 mM salt concentration. (**B**) The variation of average number (<NS_sp_> black line) and duration (<T_sp_> red line) of sliding events during the process of specific protein-DNA complex formation as functions of Φ.

The impact of crowders, however, is entirely different in the second phase, i.e. when the protein reaches the target site and is in the process of forming the specific protein-DNA contacts. Figure 3A presents the variation of sliding and hopping probabilities as a function of Φ. In this phase, 3D diffusion is restricted due to the formation of stronger specific contacts between the recognition helix of the searching protein and the target DNA sequences that prevents complete dissociation of the protein from the DNA surface. The hopping propensity, unlike the nonspecific search regime, increases with Φ and reaches the maximum at Φ = 0.2. The overall increment is by ≈13% compared to when protein diffuses in the absence of crowders. To this end, we note that the extent of increment in the propensity of hopping dynamics and the associated Φ value at which the hopping propensity is maximum rely on the nature of crowder molecules and their interactions with the protein and DNA molecules. To test this, we have adopted a model in which crowders interact with other molecules through a different potential (see Supplementary text and Fig. S4) and estimated the target search propensities both in nonspecific and specific target search regime. The search propensities are indifferent to the crowder concentration during the nonspecific search regime, whereas the hopping propensity increases by ~14% and is maximum at Φ = 0.1. The results differ slightly from the present one. Nevertheless, the underlying physics remains the same that indicates a crucial role of crowder molecules during specific contact formation.

Beyond Φ = 0.2, the hopping propensity starts decreasing. The sliding propensity shows a complementary behaviour and is minimum at Φ = 0.2. What causes this variation in the search modes during the formation of a specific protein-DNA complex? The analysis presented in Fig. 3B shows the variation of the average number of sliding events and their duration with respect to Φ. Both the quantities slightly decrease initially with increasing Φ until they reach minima at around Φ ≈ 0.2–0.3 and then start increasing again. The rationale behind this is that unlike the nonspecific search regime, where the protein smoothly diffuses within the depletion region against a fixed microscopic viscosity, the protein dynamics at the target site is different. The strong specific contacts between the protein and the DNA molecule halts the former at the target DNA site, spanned over nine base pairs. Consequently, the protein spends ≈40% time in the sliding mode in the absence of crowders. This is ≈25–30% more than that of the nonspecific search regime. Furthermore, the attenuation in the linear diffusion (due to confinement at the target DNA site) makes the searching protein vulnerable to the crowder action. Initially, the frequent collisions by the crowders promote hopping dynamics in the searching protein at the cost of a reduction in its sliding ability. This results in a dip in the average number of sliding events as well as the duration of each event at Φ ≈ 0.2–0.3. When the crowder concentration is higher (Φ > 0.3), and the corresponding depletion layer is narrower, the strong specific contacts between the protein and DNA molecules strongly confine the former inside the DNA major groove and compel it to perform sliding. Consequently, the number of sliding events and the duration per event increase once again.

### Role of crowders in regulating the mechanistic details of search dynamics

**Figure 4:**
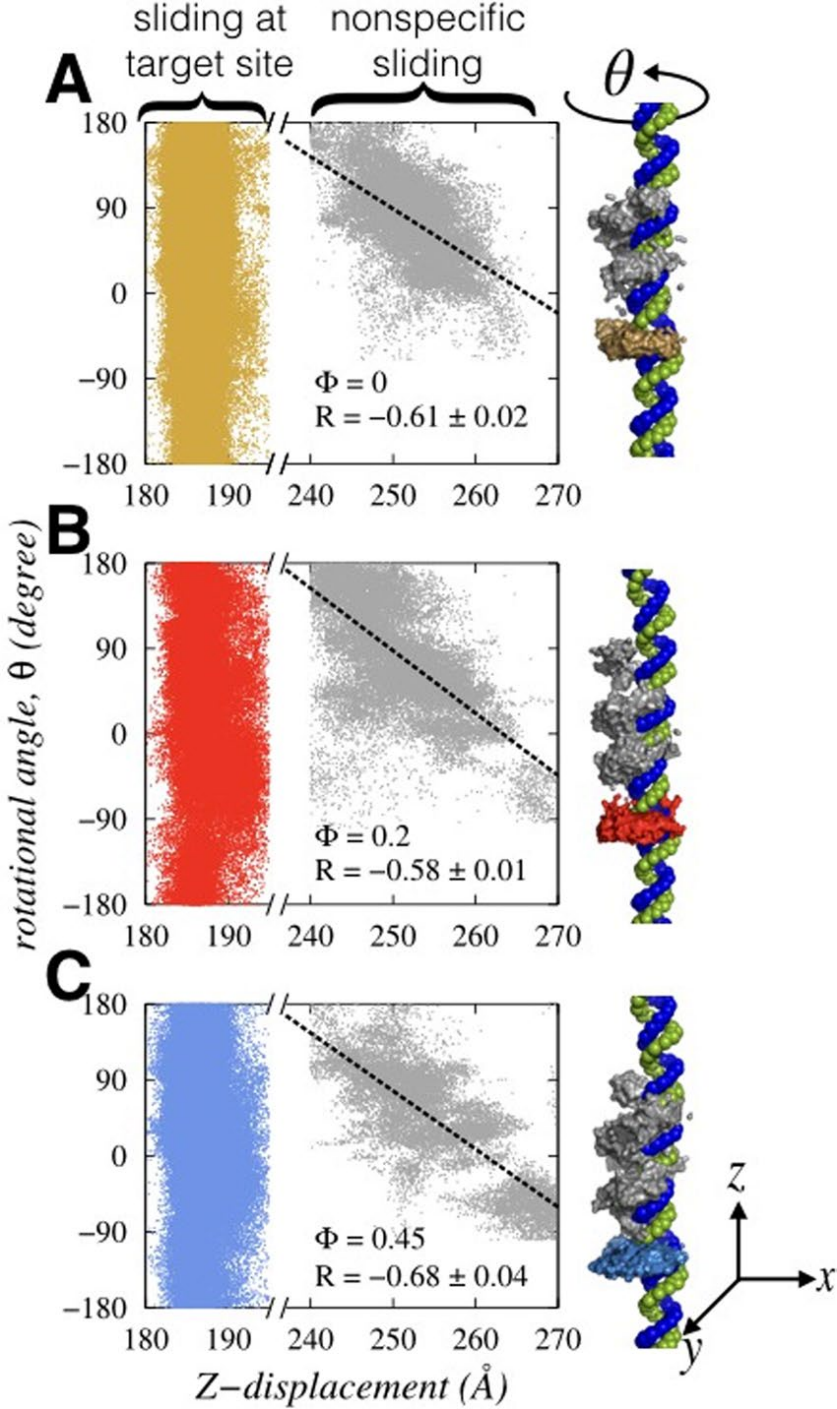
Crowder induced alterations in the sliding mechanism. The correlation between transversal (Z-axis) and rotational (θ) motions of Sap-1 are measured and presented for three different Φ values, namely (**A**) 0, (**B**) 0.2 and (**C**) 0.45. The coloured and the grey regions denote the path of sliding dynamics in specific and non-specific regime respectively. During sliding dynamics at the target site, the protein moves little along z-direction but samples a wide range of rotational angle θ to recognise the optimal orientation to form the specific protein-DNA complex. In contrast, the mechanistic details of sliding dynamics in the nonspecific region varies with crowder concentration. The correlation coefficient R denotes how the protein scans the DNA by inserting its recognition helix into the major groove of DNA. Depending on the crowder concentration, this motion could be smooth where rotation of the protein along DNA axis is coupled with the transversal motion (high R value as in Φ = 0.45). Alternatively, these two motions could be uncorrelated or only weakly correlated as well (small R, as in Φ = 0.2).

Having seen the differences in modes of transport during the specific and nonspecific search regimes with respect to crowder concentrations, we now turn to probe the mechanistic details of these search modes. Several coarse-grained models have already been employed. Most of them adopted heavily reduced descriptions of the interacting molecules and therefore lack many crucial structural details. The restraints prohibit delineating the molecular description of different search modes. For example, in our view, an adequate structural detail of DNA is an absolute must in order to differentiate between the sliding and hopping (see Supplementary text for details) modes of the searching protein. Indeed, it is observed that the nuances in major groove geometry can significantly alter the propensities of search modes^9,40,41^. To this end, we emphasize the fact that the present DNA model is chemically accurate and reproduce the sequence dependent structural and geometrical variations of the DNA molecule for varying salt and temperature condition. This encourages analyzing the mechanistic details of the search dynamics by careful scanning of the ensemble of snapshots sampled during the simulations. In Fig. 4, we monitored how the transversal displacement along the DNA contour (Z- displacement) and the ability to perform spiral motion along the DNA major grooves (rotational angle, θ) vary with the crowder concentrations when the searching protein perform sliding motion. The results show a couple of significant features corresponding to the two distinct phases of search dynamics: (1) the ability of the protein to slide differs in nonspecific target search regime depending on the cellular environment. For example, we note that in the absence of crowder molecules (Φ = 0), the transversal displacement of the protein along the DNA axis (z-axis displacement) is moderately coupled (correlation coefficient, R = −0.61 ± 0.02) with its spiral motion along the DNA major groove. Under highly dense cellular medium (Φ = 0.45), such correlation is even higher (R ≈ −0.68), owing to a narrow depletion region as discussed in Fig. 1. The protein, while scanning the DNA base pairs in sliding mode, is forced to move along the DNA by protruding its positively charged recognition helix into the spiral path (see Fig. 4) of the DNA major grooves. Typically, such concomitant rotation and advancement along the DNA contour are slow but useful as the probability of missing any DNA base pair is negligible. However, when the cellular medium is moderately crowded (Φ = 0.2), the scanning along the DNA contour is slightly less coupled (R ≈ −0.58) with the rotational motion of the protein around the DNA major grooves. The reduced correlation is because of the randomness in sliding dynamics originated due to collisions with crowder molecules that may relocate the searching protein within a relatively wider depletion layer. The biological advantage of this motion is that the protein can diffuse efficiently without getting trapped as it does not require to visit all DNA sites. Often, many sites on the genomic DNA are occupied by other DBPs. Under such cellular condition, a rotation uncoupled or weakly coupled sliding interspersed by hopping dynamics is beneficial to bypass the obstacles while searching for the target DNA site. This is reminiscent of the rotation uncoupled sliding dynamics of p53 reported by Terakawa *et al*.^42^ at a high salt condition. Similar dynamics is also noticed previously by us, where the DNA topology is found to be the factor responsible for uncoupling between the rotational and transversal motion of a searching protein^9^. (2) The second important aspect of the sliding mechanism is that, during the specific complex formation, protein’s transversal motion is strongly confined to a short patch (180 Å–190 Å along the DNA axis), where the nine base pair long target site is located. The protein, being strongly associated there, explores the best possible orientation such that the maximum number of specific contacts can be formed. This is reflected from the wide range of the rotational angles (−180^◦^ to +180^◦^) scanned by the searching protein. The feature is similar for all the three crowder concentrations, suggesting that a common mechanism of forming the specific complex may exist.

### Impacts of cellular crowding on target search kinetics of DBPs

**Figure 5:**
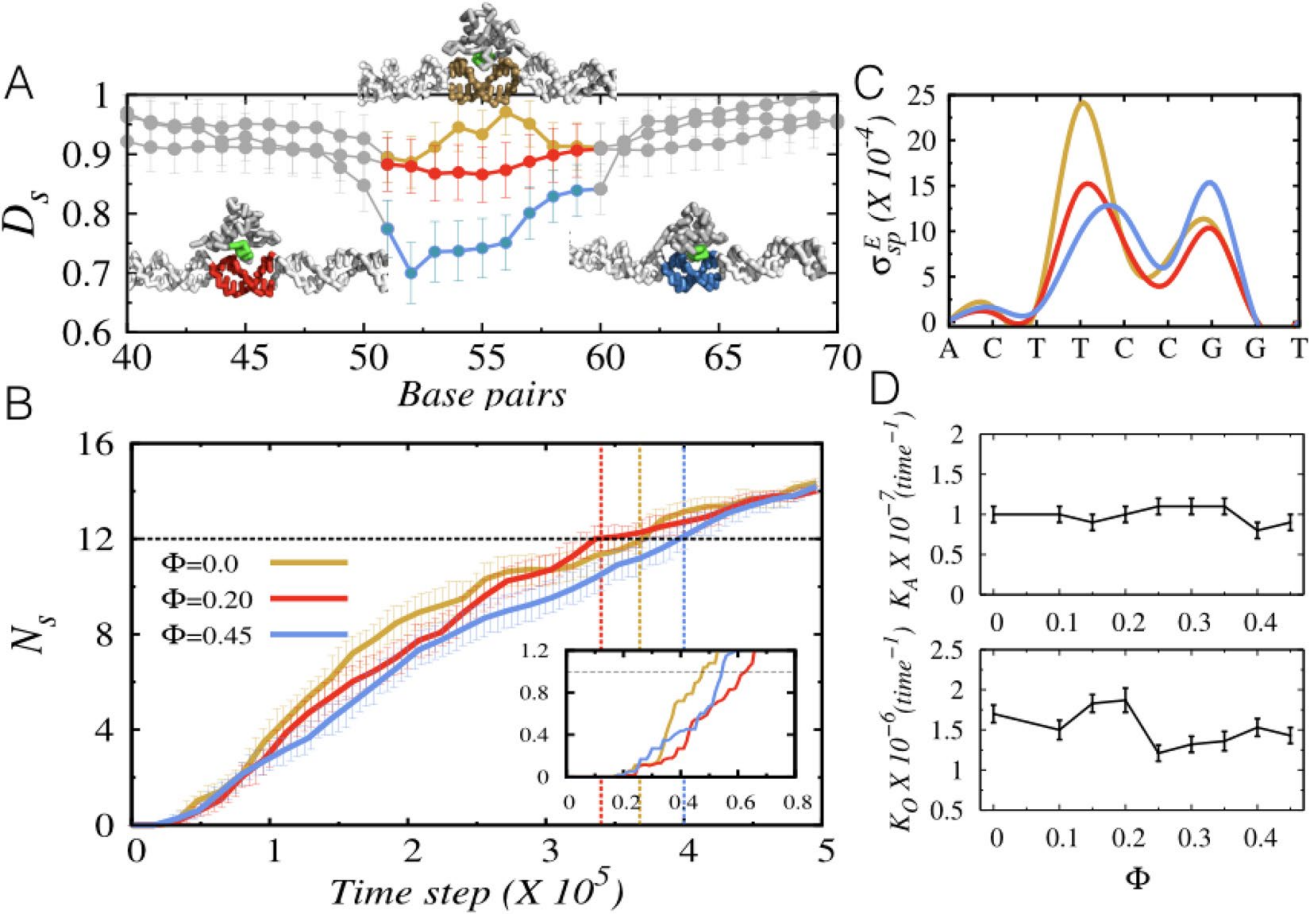
Impact of crowders on target search kinetics of DBPs. (**A**) Crowder induced deformation (Ds) per nucleotide is presented. The deformation at the target DNA site when the protein is in process of forming the specific protein-DNA complex. (**B**) The average number of specific contacts Ns as a function of simulation time for different Φ values. The kinetics for forming the first specific contact is shown in the inset. (**C**) The ruggedness per base pair (from 3′ to 5′ direction) of the specific binding energy landscape is estimated from the variance of specific energies (σ^E^_sp_). (**D**) The overall rates for the two distinct phases (as described in the text) of target search dynamics is presented as a function of crowder concentration Φ. *K*_*A*_ denotes the rate of association with which the protein reaches the target site, whereas *K*_*O*_ represents the rate with which the protein samples and find its optimal orientation for forming the most stable specific protein-DNA complex.

Upon structural analysis, we, however, observe significant differences in the bound DNA conformations for varying crowder concentrations, indicating that the latter may also regulate the specific complex formation. By analysing an ensemble of DNA structures, where the protein is specifically bound to the DNA target site, we estimate the deformation parameter, *D*_*s*_ (described in METHODS section) and present as a function of DNA base pairs in Fig. 5A. The plot indicates that the deformation in the bound state region of the DNA is much more than that of the nonspecific region (shown in grey colour). Examples of similar DNA bending, induced by interacting proteins, is abundant in nature and is known to play crucial roles in determining the enzymatic activity and specificity^43–45^. Our results, however, suggest that the deformation of the DNA conformation is not merely caused by the interacting protein, rather due to a combined effect of crowder molecules and the specific protein-DNA interactions. The synergy is further manifested by the fact that similar degree of DNA bending is absent in the nonspecific DNA region, where protein interacts with the DNA only weakly. The role of crowder molecules is strongly felt when the protein can perturb the DNA molecule through the formation of strong specific interactions. Higher the crowder concentration, more is the degree of deformation in the DNA target site. For example, the highest bending in the target site conformation is observed at Φ = 0.45, whereas a moderate bending is obtained when the crowder concentration (Φ = 0.2) is not too high. To this end, we note that a recent study has established a direct connection between nonspecific diffusion of protein and the DNA bending by arguing that a sharp bending in the DNA may disrupt the protein diffusion. The protein pauses at the bent site until the DNA eases back into a less bent conformation. This evokes an important question that if similar bending in the target DNA conformation, arising due to cellular crowding, can affect the specific binding of the protein and its kinetics? To investigate this, we perform kinetic experiments at physiological salt condition (140 mM) for three different crowder concentrations, namely, Φ = 0, 0.2, and 0.45. In Fig. 5B, we present the average number of specific contacts formed as a function of simulation time. We find that in the absence of crowders (Φ = 0), the protein is extremely quick in establishing the initial (see inset Fig. 5B) contacts. However, the specific complex formation slows down once approximately half (*N*_*s*_ ≈ 8–10) of the total number of specific contacts are formed. The reason behind the observation is that the stronger specific interactions between the recognition helix of the protein and the target DNA site present a highly rugged specific binding energy landscape (Fig. 5C and METHODS section for details). While diffusing on it, the protein may get trapped in local minima, leading to slower kinetics. Crowder molecules regulate the search process in a two-pronged manner: (1) it makes the protein hop out of these local energy minima by frequently colliding with it, provided the depletion region is wide enough to allow the protein performing hopping dynamics, (2) the collisions also change the orientation of the searching protein repeatedly and helps in recognising the optimal orientation for formation of a stable specific protein-DNA complex without being stuck in a specific orientation for a long time. An appropriate orientation is also crucial in lowering the ruggedness of the specific binding energy landscape by minimising the frustration in specific interactions. Thus, at Φ = 0.2, the ruggedness of the energy landscape is lower compared to when there is no crowder molecule (Φ = 0). The searching protein can approach the DNA better or as close as it does at Φ = 0 to stabilise the specific protein-DNA complex (as can be seen in Figs S5 and S6, the smaller or comparable distance between protein and DNA and lower *E*_*sp*_ values at Φ = 0.2 compared to Φ = 0). In combination, moderate crowder concentration enhances the kinetics of forming the specific protein-DNA complexes. For example, our result suggests that at Φ = 0.2, the time required to form ≈80% of the all specific contacts is ≈3 × 10^5^ MD steps, which is roughly 7.3% faster than when the protein binds in absence of crowders. Crowders however, do not always promise an enhancement in kinetics. Our result indicates that same ≈80% specific contact formation occurs at a much slower rate (≈17.6%) for Φ = 0.45 compared to that when Φ = 0.2. The higher concentration of crowders reduces the available conformational space for the protein by narrowing the depletion region (as discussed in Fig. 1C). As a result, the orientation process of the protein is hindered. Corresponding distance between the protein and DNA molecule (large see Fig. S5) and the specific energy (greater *E*_*sp*_ see Fig. S6) is greater compared to those in Φ = 0.2, signifying formation of a less stable specific protein-DNA complex. During the nonspecific search, the absence of strong interactions between the protein and DNA molecules offer a relatively flat binding energy landscape (see Fig. S7) on which the protein diffuses fast without much help needed from the crowders. This explains why the nonspecific target search of proteins has little dependency on the crowder molecules. Even if the nonspecific DNA segments contain patches of sequences that are close to the target DNA sequence (often referred as pseudo target sites) and the searching proteins weakly associate with it through short-ranged attractive forces, the propensities of different search mechanisms display surprising robustness (see Fig. S8). In a nutshell, the complete molecular description of the role of crowder molecules on target search of DBPs under crowded environment indicates that the time, τ_1_ required to reach the target site by the protein after sensing the DNA molecule is roughly similar for all crowder concentration. This is reflected by the association rate constant values (*k*_*A*_) in Fig. 5D that shows a steady behavior irrespective of the crowder concentration Φ. However, the time, τ_2_ required for the formation of the specific protein-DNA complex vary with Φ values. Our result suggests that there exist an optimal crowder concentration, in this case, Φ = 0.2 (0.006 w/v) that facilitates the formation of the specific protein-DNA complex. The associated sliding and hopping propensities are ≈33% and ≈67% respectively that provides the optimally weighted balance between the fast 1D search (hopping) and the accurate 1D scanning (sliding) of the DNA base pairs. The existence of optimal crowding for faster kinetics is also confirmed previously by Ge *et al*.^46^, who has studied the expression of *Renilla luciferase* driven by T7 promoter in presence of three different crowding agents, namely PEG-8000, Ficoll-70 and Ficoll-400. They observed the maximum enhancement in the transcription for Ficoll-70 at a moderate crowder concentration 0.2 (w/v) compared to its dilute solution. A similar observation was noted for Ficoll-400 as well. Our study provides the molecular basis of such observations. It is worthy to note here that unlike Ficoll, PEG-8000 exhibits an inhibitory effect on transcription process with its increasing concentration^46^ (for concentration more than 10% (w/v)). This is due to its ability to dehydrate protein surfaces that cause rapid protein precipitation before transcription. Our crowder model does not take such possibility into account at present, instead sets this as a future goal.

## Discussion

In summary, we provide a comprehensive molecular description of the role of crowder molecules in the target search dynamics of DNA binding proteins by using coarse-grained models of these biomolecules. The dynamics of crowder and DNA molecule results into formation of a depletion layer, the existence of which is supported by the observed depletion volume around a DNA molecule under *in vivo* condition commonly referred as hydration shells, formed by hydration forces. Our study reveals that the increase in crowder concentration narrows down the depletion region and at the same time enhances the crosstalk between the searching protein and the crowder molecules through frequent collisions. While the former impels the protein to stick to the DNA surface and adopt sliding as the preferred mode of translocation, the later promotes hopping dynamics. During nonspecific target search regime, where the interaction energy landscape is relatively smooth, these two competing effects nullify each other to present a negligible impact of crowder molecules on the target search dynamics. The protein, in that case, being present inside the depletion region, diffuses along the DNA contour with equal ease against a constant microscopic viscosity irrespective of the bulk crowder concentrations. The scenario, however, changes when the protein is in the process of establishing the specific protein-DNA contacts at the target DNA site. The strong specific interaction presents a rugged binding energy landscape within which the searching protein often gets trapped while diffusing. Looking into molecular level, we find that the crowder molecules aid the searching protein in a two-pronged manner. First, they collide with the protein continuously to hop it out of the local energy minima. Secondly, these frequent collisions from crowding agents help the protein to orient appropriately for the specific complex formation and thereby reduce the ruggedness of the binding energy landscape by minimising the frustrations in specific interactions. Thus, the presence of crowder molecules enhances the target search kinetics by primarily facilitating the specific protein-DNA complex formation. The degree of facilitation, nonetheless, depends on the crowder concentration and we note that the fastest target search kinetics is obtained at a moderate crowder concentration (Φ = 0.2) that tunes the width of the depletion region and the degree of protein-crowder crosstalk to their optimal values. Similar crowder concentration is reported as the preferred one for facilitated intracellular diffusion of DBPs and their activity. The protein, under such condition, orients faster and precisely to form the specific protein-DNA complex with the help of a weighted balance between sliding and hopping dynamics.

Although the presented model seems to be successful in capturing crucial insights of the protein target search in a crowded cellular milieu, one should note that our approach is still oversimplified. Many crucial phenomena of protein-DNA interactions in the crowded environment are not taken into consideration. This includes crowder induced DNA condensation (psi-condensation) that reduces the approachability of RNA polymerase and hinder the transcription. Furthermore, considering DNA fragment shorter than the persistence length (50 nm) of B-DNA filters the possibility of observing any significant deformation in the DNA structure. It will be important to extend the model to capture such complex phenomena by incorporating necessary modifications. Alongside, another important direction of future work is to probe the impacts of explicit crowders and their physical properties such as sequence, size, shape, and mobility on the target search mechanism of DNA binding proteins.

## Methods

### Protein, DNA and crowder models

Coarse-grained computational models promise great advantages in investigating complex biological processes provided they are tailored appropriately^47–49^. Here, we adopt a model similar to our previous study^9^, the salient features of which is described here briefly. The details can be found in the Supplementary text. The protein structure is represented by one bead per amino acid, placed at the respective C_α_ position. We select Sap-1 (PDB ID: 1BC8, see Fig. S9) as the searching protein. It is a single domain transcription factor that binds specifically with a nine base pair (bp) short DNA segment by protruding its helical recognition region inside the DNA major groove. The protein maintains its folded structure during the simulation, which is ensured by a structure based Lennard-Jones potential^50^. Besides, electrostatic interactions are considered through Debye–Huckel potential between the negatively charged (Glu and Asp) and positively charged (Arg and Lys) amino acids. We note that Debye-Huckel potential is an efficient way to incorporate the effects of salt concentration and thus allows us to study the salt-dependent diffusion of proteins on DNA. Although the theory has limitations and can be applied only for dilute ion concentration, it has successfully captured many crucial features of nucleic acid biophysics^51,52^. The DNA molecule is designed by adopting the 3SPN.2 model, developed by Pablo *et al*.^31^. Each nucleotide is represented by three beads located at the centres of phosphate, sugar and nitrogenous bases respectively. The model has been successful in reproducing DNA hybridisation, and estimating the persistence length and melting temperature precisely for varying composition and ionic strengths^53^. The DNA molecule is allowed to interact with the searching protein in two ways: first, the electrostatic interaction between the charged residues of protein and the phosphate beads of DNA that steer the dynamics of the protein and secondly, the excluded volume interaction that acts during the nonspecific encounter between the two biomolecules. The crowders are presented as uncharged spheres that occupy a volume fraction (Φ) determined by Φ = 4N_c_πR^3^/3L_x_L_y_L_z_, where L_x_, L_y_ and L_z_ denotes the dimensions of the simulation box with the periodic boundary condition, N_c_ is the total number of crowders and R denotes the radius of the crowder, set at 10 Å. It is noted here that crucial insights in protein-protein interactions and the thermodynamics of protein folding have been gained in the presence of macromolecular crowders^54–57^. The same for the target search kinetics of DBPs is less explored. In our simulations, crowders are allowed to interact only through nonspecific excluded volume interaction modelled by Lennard-Jones potential (see Supplementary text for details).

### Simulation Protocol

The initial structure of the 100 bp B-DNA is generated using w3DNA (3D DNA structure) web server^58^. The DNA is placed in the middle of the simulation box of size 150 Å × 150 Å × 400 Å with randomly distributed crowders and the searching protein far away from the DNA surface. The dynamics of the protein along the DNA molecule in a crowded environment was simulated using Langevin equation with friction coefficient γ = 0.05, temperature, T = 300 K and under a physiological salt concentration of 140 mM. Each simulation is 1 × 10^8^ MD steps long and at least 20 such independent simulations are done for each system with varying Φ in order to achieve significant statistics. We also perform kinetic experiments, which are of 5 × 10^7^ MD steps longer. For each Φ value, we perform 300 such simulations to investigate the kinetics of the search process. During the kinetic experiment, we study the formation of the specific protein-DNA complex. The specific interactions are incorporated in the present model by inserting the nine bp target DNA sequence as found in the crystal structure of Sap-1, at the centre of a 100 bp DNA sequence. Initially, the protein is placed closed to DNA surface but far away from the target site. The specific contacts between the recognition helix of sap-1 and the target DNA site are identified and modelled through a short-range Lennard–Jones potential.

### Analysis

The identification of different search modes, such as sliding, hopping and 3D diffusion is done by following the prescription of our previous work and is also described in the text and Fig. S10 of Supplementary Material. Briefly, the protein performs 3D diffusion in bulk if it is far away (more than 30 Å) from the DNA surface. In contrast, the protein scans the DNA base pairs through sliding dynamics if it is close to DNA and is oriented such that its recognition helix stays mostly inside the major groove. Hopping is confirmed in a snapshot if the protein is close enough to the DNA but does not satisfy any one of the criteria for sliding. Therefore, when the protein is searching the DNA nonspecifically, it can either slide or hop along the DNA. This indicates that these two modes of motion are complementary to each other. Increasing the propensity of one lowers the propensity of other mode. In the analysis, we present a parameter *D*_*s*_ that captures the degree of DNA deformation. To estimate this, we determine the local coordinates from the centre of each DNA base pairs. Deformation per nucleotide is defined as the average difference between the local coordinate of one base pair and its reference position in the ideal B-DNA geometry. *D*_*s*_ denotes the relative value of such deformations, normalised with respect to the maximum deformation of DNA observed by sampling an ensemble of DNA conformations at different Φ values. We also estimate the ruggedness of protein-DNA binding energy landscape. The ruggedness per nucleotide position, σ^E^_sp_, is simply measured from the corresponding fluctuations in excluded volume energies for all DNA sites and specific energies for the target site region only.

## Electronic supplementary material

Supplementary Information
