# Supplementary material for: Role of Macromolecular Crowding on the Intracellular Diffusion of DNA Binding Proteins
Source: Sci Rep. 2018 Jan 16;8:844. doi: 10.1038/s41598-017-18933-3 (PMC5770392; doi:10.1038/s41598-017-18933-3)
Supplement: Supplementary file 1 — Supplementary Information [file 41598_2017_18933_MOESM1_ESM.pdf]

Supplementary for:

Role of Macromolecular Crowding on the Intracellular Diffusion of DNA Binding Proteins

Pinki Dey and Arnab Bhattacherjee*

School of Computational and Integrative Sciences, Jawaharlal Nehru University, New Delhi-110067, India

E-mail: arnab@jnu.ac.in

Contents

Protein-DNA-Crowder model

System Preparation

Criteria of searching modes

Tables

References

Figures

Protein-DNA-Crowder Model:

Protein Model:

The protein selected here is Sap-1 (PDB ID: 1BC8). It is a 93-reside single domain transcription factor that binds specifically with a 9-base pair (bp) short DNA segment by protruding its helical recognition region inside the DNA major groove. The protein maintains its folded structure during the simulation, which is ensured by a structure based Leonard-Jones potential[1]. The same model has been used extensively by us and other groups to study protein-DNA interactions and various aspects of protein folding[2–7]. Besides, electrostatic interactions are considered through Debye–Hűckel potential between the negatively charged (Glu and Asp) and positively charged (Arg and Lys) amino acids. We note that Debye-Hűckel potential is an efficient way to incorporate the effects of salt concentration and thus allows us to study the salt-dependent diffusion of proteins on DNA. Previous study has adopted the same to successfully capture many crucial features of nucleic acid biophysics[2,6]. However, one should be aware of its approximations. The model is valid for relatively dilute solutions of monovalent ions and does not take ion condensation into

consideration. In particular, it fails[8] for an ion concentration > 0.5 M.

The potential energy of protein molecule is given as

$$E_{pot} = E_{bond} + E_{bend} + E_{torsion} + E_{LJ} + E_{ev} + E_{ele} \quad (1)$$

E_{bond} represents bonded energy and is given by,

$$E_{bond} = \sum_i k_b (r_i - r_i^0)^2 \quad (2)$$

where k_b =100.0 kcal/mol/Å^2 , r_i and r_i^o are the distances between i-th and i+1-th C_α beads in intermediate and folded structures respectively.

E_{bend} is the potential energy function for variation of angles and is given by,

$$E_{bend} = \sum_i k_\theta (\theta_i - \theta_i^0)^2 \quad (3)$$

where k_θ = 20.0 kcal/mol/rad^2, θ_i and θ_i^0 are the angles among i-th, i+1-th, i+2-th C_α beads in intermediate and folded structures respectively.

$E_{torsion}$ is the potential energy function for torsional angle formed by every four atoms connected by chemical bonds and is given by,

$$E_{torsion} = \Sigma_i \{ k_{\phi 1}[1 - cos3(\phi_i - \phi_i^0)] + k_{\phi 2}[1 - cos(\phi_i - \phi_i^0)] \} \quad (4)$$

where $k_{\phi 1}$=0.5 kcal/mol, $k_{\phi 2}$=1.0 kJ/mol, ϕ_i and ϕ_i^0 are the torsional angle among i-th, i+1-th, i+2-th, i+3-th C_α beads in intermediate and folded structures respectively.

E_{LJ} is the conformational energy that is estimated by using a native topology based model[2] in which a Lennard–Jones potential favoured the formation of contacts found in folded structure of the protein.

$$E_{LJ} = \Sigma_{i<j-3}^{native} \epsilon_{ij} \left[5 \left(\frac{\sigma_{ij}}{r_{ij}} \right)^{12} - 6 \left(\frac{\sigma_{ij}}{r_{ij}} \right)^{10} \right] \quad (5)$$

where ε_{ij} =3.824091778 kcal/mol, r_{ij} represents the distance between native pairs at a given time and σ_{ij}=4.0 Å, denotes the closest possible distance between two interacting C_α beads.

E_{ev} denotes the excluded volume effect between all non-bonded and non-native pairs of protein. The explicit form is given by,

$$E_{ev} = \Sigma_{i<j-3}^{non-native} \varepsilon_{ev} \left(\frac{\sigma_{ij}}{r_{ij}} \right)^{12} \quad (6)$$

where ε_{ev} =0.239 kcal/mol, r_{ij} is the distance between i-th and j-th beads and $\sigma_{ij} = \sigma_i + \sigma_j$ is the interaction specific length scale, where σ_i and σ_j are radii of respective interacting beads.

E_{ele} is the electrostatic potential energy function and is represented by Debye-Hűckel potential as,

$$E_{elec} = \sum_{i<j} \frac{q_i q_j e^{-r_{ij}/\lambda_D}}{4\pi\epsilon_0 \epsilon(T,C) r_{ij}} \quad (7)$$

where q_i and q_j are the charges of sites i and j, r_{ij} is the inter site separation, λ_D is the Debye screening length, ϵ(T, C) is the dielectric permittivity of the solution. The Debye screening length is defined as,

$$\lambda_D = \sqrt{\frac{\epsilon_0\epsilon(T,C)}{2\beta N_A e_c^2 I}}$$

where β is the inverse thermal energy of the system $(K_B T)^{-1}$, N_A is Avogadro's number, and I is the ionic strength of the solution. The solution dielectric permittivity ϵ(T, C) is a function of the molarity of NaCl and temperature as below[9]:

$$\epsilon(T,C) = \epsilon(T)a(C) \quad (8)$$

where,

$$\epsilon(T) = 249.4 - 0.788\,T/K + 7.20\times10^{-4}(T/K)^2 \quad (9)$$

and

$$a(C) = 1.00 - 2.551\,C/M + 5.151\times10^{-2}(C/M)^2 - 6.889\times10^{-3}(C/M)^3 \quad (10)$$

DNA model:

We adopted 3SPN.2 model developed by Pablo et. al[9] to describe the DNA force field. Each nucleotide is represented by three beads located at the centres of phosphate, sugar and nitrogenous bases respectively. The model provides good agreement with experimental measures of structural properties such as duplex width, base rise, and major and minor groove width. It captures the persistence length of both ss- and dsDNA and predicts melting temperatures that are consistent with experiment. Furthermore, the model precisely captures the hybridization and reproduce the dynamics of dsDNA in solution.

In 3SPN.2 model, the potential energy function as follows:

$$E_{pot}^{DNA} = E_{bond}^{DNA} + E_{bend}^{DNA} + E_{tors}^{DNA} + E_{exe}^{DNA} + E_{bstk}^{DNA} + E_{cstk}^{DNA} + E_{bp}^{DNA} + E_{elec}^{DNA}$$

E_{bond}^{DNA} is the potential energy function for covalent bonding interactions and is given by,

$$E_{bond}^{DNA} = \sum_i k_b\left(r_i - r_i^0\right)^2 + 100k_b\left(r_i - r_i^0\right)^4 \quad (11)$$

where k_b= 0.6 kJ/mol/Å^2 and r_i, r_i^0 are respectively the instantaneous and equilibrium bond length for the i-th bond.

E_{bend}^{DNA} is the potential energy function for molecular bending and is given by,

$$E_{DNA}^{bend} = \sum_i k_\theta (\theta_i - \theta_i^0)^2 \tag{12}$$

Where k_θ = 200 kJ/mol/rad^2, θ_i, θ_i^0 are respectively the instantaneous and equilibrium bond angles for the i-th bond angle.

E_{tors}^{DNA} is the potential energy function for torsional angle formed by every four atoms connected by chemical bonds and is given by

$$E_{tors}^{DNA} = \sum_i - k_\phi \exp\left(\frac{-(\phi_i - \phi_i^0)^2}{2\sigma_{\phi,i}^2}\right) \tag{13}$$

where k_ϕ = 6.0 kJ/mol, ϕ_i, ϕ_i^0, and $\sigma_{\varphi,i}$denote the well-depth, equilibrium angle, and Gaussian well-width, respectively, of dihedral *i*.

E_{exe}^{DNA} denotes the excluded volume of purely repulsive potential between sites *i* and *j* of the form,

$$E_{exe}^{DNA} = \sum_{i<j} \begin{cases} \epsilon_r \, [(\frac{\sigma_{ij}}{r_{ij}})^{12} - 2(\frac{\sigma_{ij}}{r_{ij}})^6] + \varepsilon_r, r < r_c \\ 0, \qquad\qquad\qquad\qquad r \geq r_c \end{cases} \tag{14}$$

where ϵ_r=1.0 kJ/mol is the energy parameter for excluded volume interactions, σ_{ij} is the average site diameter, and r_{ij} is the inter site separation. r_c is the cutoff distance.

E_{bstk}^{DNA} is the potential energy function for intra-stand Base-stacking and is given by,

$$E_{bstk}^{DNA} = \sum_{bstk} \begin{cases} U_m^{rep}(\epsilon_{ij}, \alpha_{BS}, r_{ij}) + (K_{BS}, \Delta\theta_{BSij}) U_m^{attr}(\epsilon_{ij}, \alpha_{BS}, r_{ij}) r_{ij} < r_{ij}^0 \\ f(K_{BS}, \Delta\theta_{BSij}) U_m^{attr}(\epsilon_{ij,}\alpha_{BS}, r_{ij}) \qquad\qquad r_{ij} \geq r_{ij}^0 \end{cases} \tag{15}$$

where K_{BS}= 6.0, α_{BS}=3.0 and $\epsilon_{ij}$is the depth of the well of the attraction between sites i and j, $r_{ij}^0$is the equilibrium separation between them, and α_{BS} is a parameter that is adjusted to control the range of attraction. The decomposition of the attractive and repulsive portions of the Morse potential is obtained by modulating the attraction using *f.* This decomposition ensures that the repulsive character is maintained, regardless of the modulating angle. Base stacking interactions are modulated by *f* using θ_{BS} .

E_{bp}^{DNA} is the potential energy function for base-pairing and is given by,

$$E_{bp}^{DNA} = \sum_{bp} \begin{cases} U_m^{rep}(\epsilon_{ij}, \alpha_{BP}, r_{ij}) + \frac{1}{2}(1 + cos(\Delta\phi_1)) f(K_{BP}, \Delta\theta_{1ij}) f(K_{BP}, \Delta\theta_{2ij}) U_m^{attr}(\epsilon_{ij}, \alpha_{BP}, r_{ij}) r_{ij} < r_{ij}^0 \\ \frac{1}{2}(1 + cos(\Delta\phi_1)) f(K_{BP}, \Delta\theta_{1ij}) f(K_{BP}, \Delta\theta_{2ij}) U_m^{attr}(\epsilon_{ij}, \alpha_{BP}, r_{ij}) \qquad r_{ij} \geq r_{ij}^0 \end{cases} \tag{16}$$

where K_{BP}=12.0, α_{BP}=2.0 and ϵ_{ij} is the depth of the well of the attraction between sites i and j, r_{ij}^0 is the equilibrium separation between them, and α_{BP} is a parameter that is adjusted to control the

range of attraction. The decomposition of the attractive and repulsive portions of the Morse potential is obtained by modulating the attraction using f, $\Delta\phi_1=\phi_1-\phi_1^0$ is penalizing deviations from a reference dihedral angle. This decomposition ensures that the repulsive character is maintained, regardless of the modulating angle. Base pairing interactions are modulated by f using θ_1 and θ_2 respectively.

E_{cstk}^{DNA} is the potential energy function for cross-stacking and is given by,

$$E_{cstk}^{DNA} = \sum_{cstk} f(K_{BP}, \Delta\theta_{3ij}) f(K_{CS}, \Delta\theta_{CSij}) U_m^{attr}(\epsilon_{ij}, \alpha_{CS}, r_{ij}) \tag{17}$$

where K_{CS}= 8.0, α_{CS}= 4.0 and ϵ_{ij} is the depth of the well of the attraction between sites i and j. The cross-stacking interaction is modulated using both $\theta_3$and θ_{CS}. Only the attractive component of the Morse potential is modulated.

E_{elec}^{DNA} is the potential energy function a screened electrostatic potential between all other inter-strand phosphates and all intra-strand phosphates not on neighboring nucleotides. This interaction is modeled by the potential energy function presented in Eqn. (7).

Protein DNA Interactions:
The model assumes two kinds of protein DNA interactions, namely non-specific and specific.

Non-specific protein DNA interactions:
During non-specific interaction the DNA molecule is allowed to interact with the searching protein in two ways: first, the electrostatic interaction between the charged residues of protein and the phosphate beads of DNA that steer the dynamics of the protein and secondly, the excluded volume interaction that acts during the nonspecific encounter between the two biomolecules. The electrostatic interaction is modeled by Debye-Hűckel potential and is given by equation (7). The excluded volume interaction is modeled by equation (6). One should note that this differs slightly from equation (14), which represents the excluded volume interaction within the DNA beads. Similar model has been adopted previously to depict the target search mechanism of DNA binding proteins[2,10].
In addition, studies suggest that often DNA sequence mediated interactions may hinder the 1D diffusion of the searching protein. Typically, this happens while protein searches DNA patches, the sequences of which share high degree of similarity with that of the target DNA site. Nonetheless, such interactions are transient and in the present study we show that even in the presence of these non-specific interactions between the protein and the non-specific DNA sequences the impact of crowder molecules remains the same (see Fig. S8). We adopted a Lennard-Jones potential to describe this DNA sequence mediated non-specific interactions with the protein molecule.[2,11]

During such interactions, the DNA bases could randomly interact with protein residues, belonging to the recognition region. Associated interaction strength ϵ_{ij} was chosen from a Gaussian distribution[12],

$(F(\epsilon) = (1/2\pi\sigma^2)^{1/2})\exp[-((\epsilon - \epsilon_{nonspecific})^2/2\sigma^2)])$ with mean (ϵ_{ij})= 0.1 and standard deviation (σ) of 0.01.

The corresponding potential energy function is given by,

$$E_{LJ} = \Sigma_{i<j} \begin{cases} k_{nsp}\left[5\left(\frac{\sigma_{ij}}{r_{ij}}\right)^{12} - 6\left(\frac{\sigma_{ij}}{r_{ij}}\right)^{10}\right], r < r_c \\ 0, \qquad r \geq r_c \end{cases} \tag{18}$$

where k_{nsp}= 0.1 kcal/mol, σ_{ij} is the average site diameter, and r_{ij} is the inter site separation. r_c is the cutoff distance set at 15 Å. Notably, the cutoff distance is such that the protein reads the DNA bases only during sliding dynamics.

Specific protein DNA interactions:

The protein upon reaching at the target DNA site can form the specific contacts. The interacting protein and DNA residues that are involved in forming the specific contacts are identified by analyzing the X-ray Crystallographic structure of the complex (PDB ID- 1BC8.pdb). A pair of protein residue and a DNA base is said to be involved in specific interactions if the distance between any two atoms of the interacting pairs is less than 3.5 Å. The formation of the specific contacts is favored by a short-ranged Lennard-Jones potential given as,

$$E_{LJ} = \Sigma_{i<j} \begin{cases} k_{sp}\left[5\left(\frac{\sigma_{ij}}{r_{ij}}\right)^{12} - 6\left(\frac{\sigma_{ij}}{r_{ij}}\right)^{10}\right], r < r_c \\ 0, \qquad r \geq r_c \end{cases} \tag{19}$$

where k_{sp}= 0.5 kcal/mol, σ_{ij} are the distances between interacting beads involved in forming the specific contacts as identified from the crystal structure of Sap-1 bound with target DNA sequences. r_{ij} is the inter site separation. r_c is the cutoff distance set at (σ_{ij}+5) Å. It should be noted that equation (18) and equation (19) are identical. However, the strength of the specific contacts is five times higher than that of non-specific sequence based protein DNA interactions, ensuring higher stability of the specific complex formation. It is also noteworthy that unlike the uniform 15 Å cutoff distance used in nonspecific interactions, here, the cutoff distances are specific to the pair of interacting beads that are involved in forming the specific contacts between protein and DNA molecules. An analysis of the crystal structure reveals that the distances between such interacting pairs range from ~12.5 Å to ~5.6 Å and therefore, the mentioned criterion allows forming specific interactions only when the protein closely reads the specific DNA bases. A larger and uniform cutoff distance for all the specific contacts may result in a perturbed dynamics of the searching protein by forcefully binding it to the specific DNA site even when the protein is not very close to the DNA or oriented differently.

Crowder model:

The crowders are presented as uncharged spheres that occupy a volume fraction (Φ) determined by $\phi = 4N_c\pi\, R^3/3\, L_xL_yL_z$, where L_x, L_y, and L_z denote the dimensions of the simulation box with the periodic boundary condition, N_c is the total number of crowders and R denotes the radius of the crowder, set at 10Å. The uncharged spherical crowders are randomly placed within the simulation box and are allowed to interact with other crowder molecules as well as protein and DNA molecules through excluded volume interaction modeled by Lennard-Jones potential given as[13],

$$E_{LJ} = \Sigma_{i<j}\begin{cases} k_{ev}\left[\left(\frac{\sigma_{ij}}{r_{ij}}\right)^{12} - 2\left(\frac{\sigma_{ij}}{r_{ij}}\right)^{6} + 1\right], r < r_c \\ 0, \qquad\qquad\qquad\qquad\qquad r \geq r_c \end{cases} \tag{20}$$

where k_{ev} =0.239005736 kcal/mol is the energy parameter for excluded volume interactions, $\sigma_{ij} = \sigma_i + \sigma_j$ is the interaction specific length scale, where σ_i and σ_j are radii of respective interacting beads and r_{ij} is the inter site separation. r_c is the cutoff distance set at $2^{1/6}\sigma_{ij}$ Å that confirms the interactions are short-ranged and repulsive in nature and therefore, the possibility of long-range interactions that can be seen for charged crowders has not been taken into consideration.

System Preparation:

Coarse-grained structure of the protein is built using atomic coordinates provided in the crystal structure with PDB ID: 1BC8. The initial structure of 100bp DNA sequence for simulations is obtained from w3DNA (3D DNA structure) web server (http://w3dna.rutgers.edu). The DNA is placed in the middle of the simulation box of size 150 Å X 150 Å X 400 Å with randomly distributed crowders and the searching protein far away from the DNA surface. The dynamics of the protein along the DNA molecule in a crowded environment was simulated using Langevin equation with frictional coefficient, γ = 0.05, temperature, T=300K and under a physiological salt concentration of 140 mM. Each simulation is 1×10^8 MD steps long and at least 20 such independent simulations are done for each system with varying ϕ in order to achieve significant statistics.

We also perform kinetic experiments, which are of 5×10^7 MD steps longer. For each ϕ value, we perform 150 such simulations to investigate the kinetics of the search process. During the kinetic experiment, we study the formation of the specific protein-DNA complex. The specific

interactions are incorporated in the present model by inserting the nine bp target DNA sequence as found in the crystal structure of sap-1, at the centre of a 100 bp DNA sequence. Initially, the protein is placed closed to DNA surface but far away from the target site. The specific contacts between the recognition helix of sap-1 and the target DNA site are identified and modeled through a short-range Lennard–Jones potential.

Criteria to differentiate various search modes:

The criteria for Sliding, hopping and 3D diffusion were followed from the methods prescribed in previous works[2–4] and is discussed briefly here (see fig S2). It is assumed that protein molecule performs 3D diffusion if the center of its recognition helix was more than 30 Å away from the center of closest DNA base pair. A snapshot is defined as sliding mode if at least 70% of recognition region is in contact with DNA major groove, the center of mass of recognition region is within 18 Å from the center of closest DNA base pair and orientation angle (θ) is <25°. If recognition helix was found at a distance of less than 30 Å from DNA and yet did not match any of the sliding criteria, the protein is considered to perform hopping along DNA. The 1D diffusion coefficient D1 is measured from linear behavior of mean square displacement of Sap-1 along DNA contour while performing sliding and hopping only.

Amino acid masses and radii:

Amino acid (single-letter code)	Mass (Da)	Radius of C_α atom (Å)
Isoleucine (I)	131.1	2.0
Lysine(K)	146.1	2.0
Phenylalanine(F)	165.2	2.0
Threonine(T)	119.1	2.0
Tryptophan(W)	204.2	2.0
Valine(V)	117.1	2.0
Arginine(R)	174.2	2.0
Histidine (H)	155.1	2.0
Alanine(A)	89.0	2.0

Asparagine(N)	132.1	2.0
Leucine (L)	131.1	2.0
Methionine(M)	149.2	2.0
Aspartic Acid (D)	133.1	2.0
Cytosine (C)	121.1	2.0
Glutamic acid (E)	147.1	2.0
Glutamine (Q)	146.1	2.0
Glysine (G)	75.0	2.0
Proline (P)	115.1	2.0
Serine (S)	105.0	2.0
Tyrosine(Y)	181.1	2.0

Masses and radii for nucleic acid components:

	Mass (Da)	Radius(Å)
Phosphate	94.97	2.25
Sugar	83.11	3.20
Adenine(A)	134.1	2.70
Thymine(T)	125.1	3.55
Guanine(G)	150.1	2.45
Cytosine(C)	110.1	3.20

References:

1. Clementi, C., Nymeyer, H. & Onuchic, J. N. Topological and energetic factors: what determines the structural details of the transition state ensemble and 'en-route' intermediates for protein folding? An investigation for small globular proteins. *J. Mol. Biol.* **298,** 937–53 (2000).
2. Mondal, A. & Bhattacherjee, A. Searching target sites on DNA by proteins: Role of DNA dynamics under confinement. *Nucleic Acids Res.* **43,** 9176–9186 (2015).
3. Bhattacherjee, A. & Levy, Y. Search by proteins for their DNA target site: 1. The effect of DNA conformation on protein sliding. *Nucleic Acids Res.* **42,** 12404–12414 (2014).
4. Bhattacherjee, A. & Levy, Y. Search by proteins for their DNA target site: 2. The effect of DNA conformation on the dynamics of multidomain proteins. *Nucleic Acids Res.* **42,** 12415–12424 (2014).
5. Bhattacherjee, A., Krepel, D. & Levy, Y. Coarse-grained models for studying protein diffusion along DNA. *Wiley Interdiscip. Rev. Comput. Mol. Sci.* **6,** 515–531 (2016).
6. Azia, A. & Levy, Y. Nonnative Electrostatic Interactions Can Modulate Protein Folding: Molecular Dynamics with a Grain of Salt. *J. Mol. Biol.* **393,** 527–542 (2009).

7. Levy, Y., Onuchic, J. N. & Wolynes, P. G. Fly-casting in protein-DNA binding: Frustration between protein folding and electrostatics facilitates target recognition. *J. Am. Chem. Soc.* **129,** 738–739 (2007).
8. Li, R., Ge, H. W. & Cho, S. S. Sequence-dependent base-stacking stabilities guide tRNA folding energy landscapes. *J. Phys. Chem. B* **117,** 12943–12952 (2013).
9. Hinckley, D. M., Freeman, G. S., Whitmer, J. K. & De Pablo, J. J. An experimentally-informed coarse-grained 3-site-per-nucleotide model of DNA: Structure, thermodynamics, and dynamics of hybridization. *J. Chem. Phys.* **139,** (2013).
10. Terakawa, T., Kenzaki, H. & Takada, S. P53 searches on DNA by rotation-uncoupled sliding at C-terminal tails and restricted hopping of core domains. *J. Am. Chem. Soc.* **134,** 14555–14562 (2012).
11. Givaty, O. & Levy, Y. Protein Sliding along DNA: Dynamics and Structural Characterization. *J. Mol. Biol.* **385,** 1087–1097 (2009).
12. Marcovitz, A. & Levy, Y. Weak frustration regulates sliding and binding kinetics on rugged protein-DNA landscapes. *J. Phys. Chem. B* **117,** 13005–13014 (2013).
13. Ma. Y, Chen. Y, Yu. W & Luo. K, How nonspecifically DNA-binding proteins search for the target in crowded environments. *J. Chem. Phys.* **144,** 125102–8 (2016).

FIGURES:

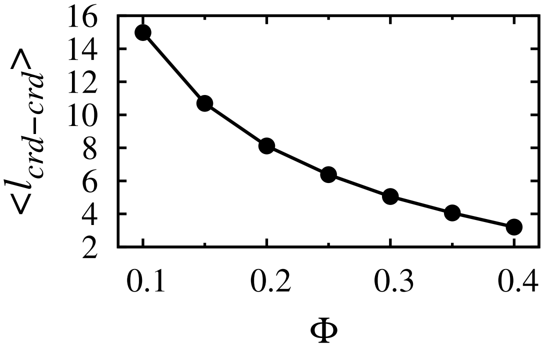

Figure S1: Variation in the average of the shortest crowder-crowder distances as a function of ϕ. With increasing crowder concentration, the inter-crowder distance decreases, which is however, quite different compared to the average of the shortest crowder-DNA distances (l_d). Had the depletion region originates due to the site diameter of interacting beads (σ_{ij} in Eqn. 20 in Supplementary text), the depletion region between crowder-DNA would have always been narrower than the crowder-crowder depletion region due to the smaller radius of phosphate beads in DNA compared to crowder molecules. This confirms the role of crowder-DNA dynamics in forming the depletion zone between crowder and DNA.

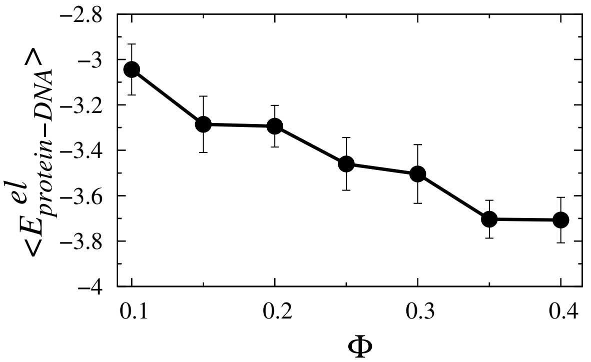

Figure S2: Variation in the average electrostatic interactions between protein and DNA molecules as a function of ϕ. Our results suggest that with increasing crowder concentration, the depletion region narrows down and therefore, the crowder molecules push the protein to stay close to the DNA surface. The present analysis confirms that the narrowing down of the depletion layer due to increased crowder concentration (ϕ) results in an increment in the electrostatic attraction between the protein and DNA molecules, which typically promotes the sliding dynamics in the searching protein.

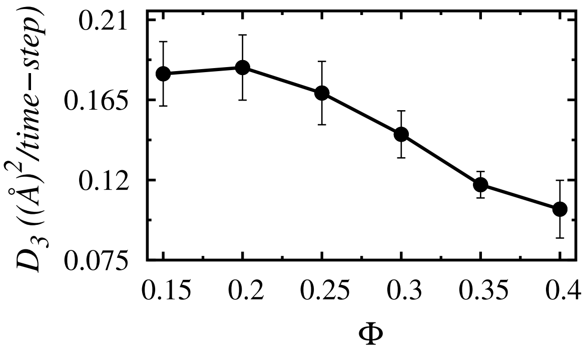

Figure S3: Variation of 3D-diffusion coefficient (D_3) as function of ϕ. The gradual decrease in D_3 signifies that with the increasing crowder concentration, protein dynamics in the bulk solution decreases.

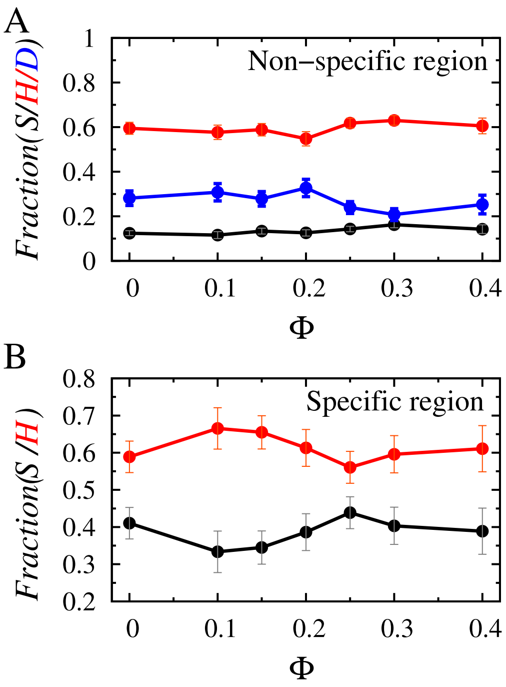

Figure S4: Effect of ϕ on the interplay between Sliding (S), Hopping (H), 3D-diffusion (D), in the specific and non-specific target search regimes, where the interaction of the crowders with itself as well as other particles is modeled by a potential given in Eqn 6. Unlike the potential used previously the present form of the potential is purely repulsive in nature and does not consider overlaps of crowder molecules. (A) The interplay between non-specific search dynamics, (S) and (H) and (D) at 140mM salt concentration. (B) The interplay between specific search dynamics, (S) and (H) at 140mM salt concentration. The similarity of the result with that of Fig 2 in the main text confirms that the underlying physics that governs the impact of crowder molecules on the target search dynamics of DBPs remains the same.

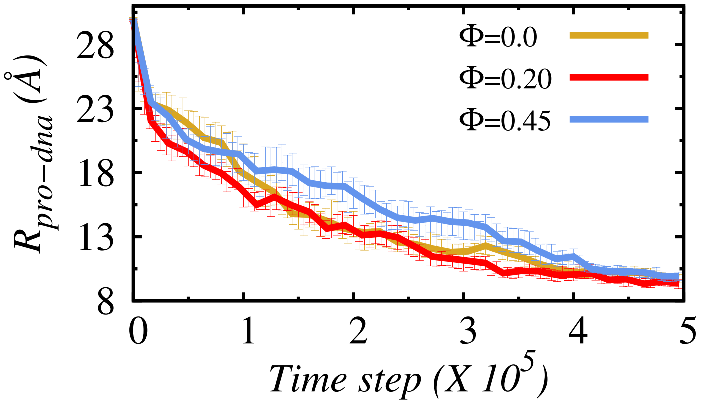

Figure S5: The figure presents the evolution of distances ($R_{pro\text{-}DNA}$) between the center of recognition region of Sap-1 and the center of the closest DNA base pair for different ϕ as a function of time.

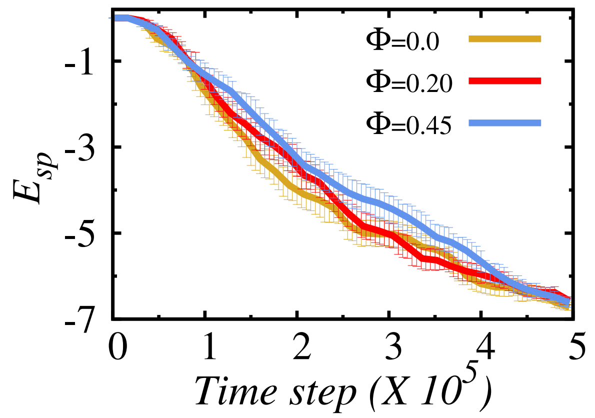

Figure S6: This figure presents the specific energies (E_{sp}) as a function of simulation time for three different volume fractions of crowders (ϕ).

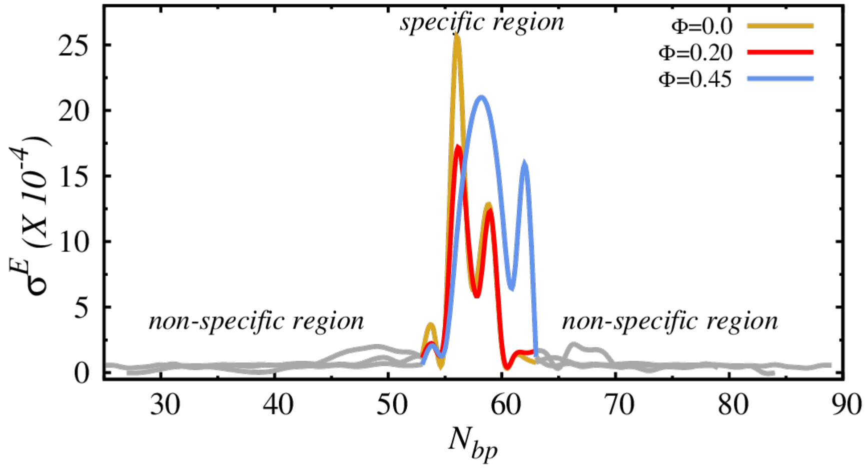

Figure S7: The figure represents the ruggedness in the energy landscape quantified by the variance in interaction energy between Sap-1 and DNA molecules in both non-specific and specific search regimes. The grey region indicates the non-specific search regime, whereas the colored region represents the specific search regime corresponding to different ϕ.

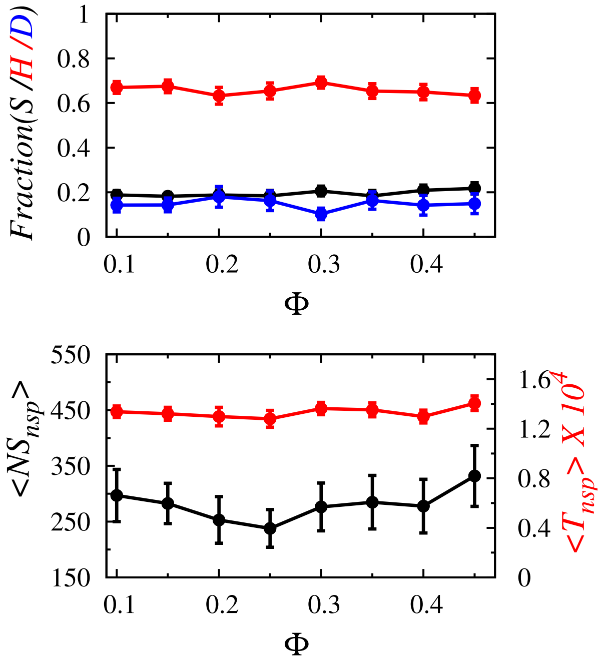

Figure S8: Effect of ϕ on the interplay between Sliding (S), Hopping (H), 3D-diffusion(3D), in the non-specific region, where DNA bases could randomly interact with the protein residues outside the target region. The strength of nonspecific interactions are however, five times weaker than that of specific interactions in order to avoid confinement of the searching protein at a random nonspecific site. Further details are given in the **Protein-DNA-Crowder** section of this document. (A) The interplay between non-specific search dynamics, (S) and (H) and (D) at 140mM salt concentration. (B) The variation of average number of non-specific sliding events (NS_{sp}, denoted by black line) and the average time spent during these sliding events (T_{sp}, denoted by red line) as a function of ϕ. The result clearly indicates that the crowder molecules have marginal impacts (uniform probabilities of performing sliding events and number of such events over various range of crowder concentrations) on the nonspecific target search mechanism, where the protein residues only transiently read out the nonspecific DNA bases.

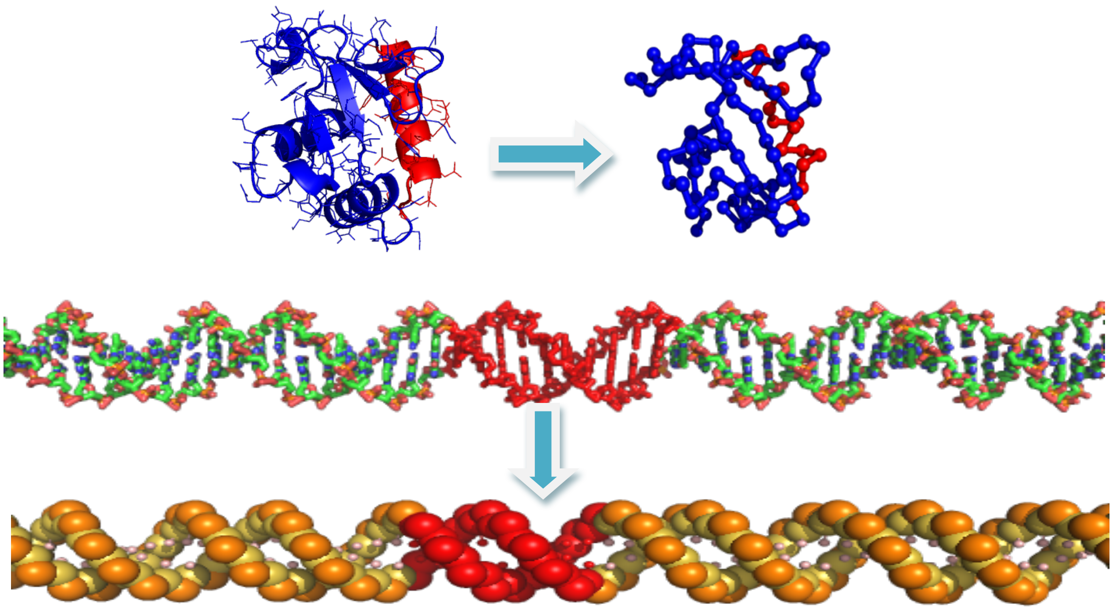

Figure S9: The overall structure of Sap-1 and DNA molecules in all atom (top left and middle) and coarse-grained (top right and bottom) representations. The recognition region for Sap-1 is labeled with red colour that corresponds to 53-68 residue region in Sap-1. Each nucleotide in DNA is presented through three beads namely; negatively charged phosphate beads (orange colour), yellow sugar beads and the small pink coloured base beads. The target site region in the DNA is represented with red colour.

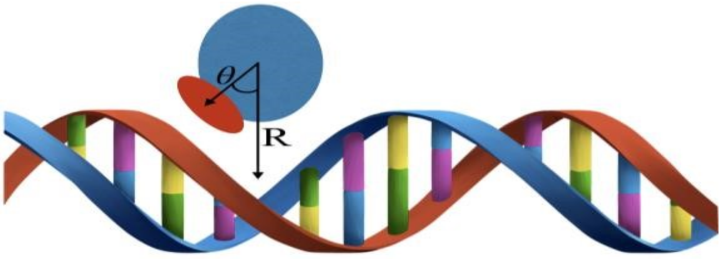

Figure S10: Schematic representation to describe various searching mechanisms, namely sliding, hopping and 3D diffusion. The protein is assumed to perform 3D diffusion if center of its recognition helix (brown colored ellipse) (R) is more than 30 Å away from center of closest DNA base pair. A snapshot is defined to be in sliding mode if at least 70% of the protein recognition region is in contact with the DNA major groove, R is within 18 Å from the center of closest DNA base pair and orientation angle (Θ) is <25°. If the recognition region is found at R<30 Å but yet did not match any of the sliding criteria, then it is considered as hopping along DNA.

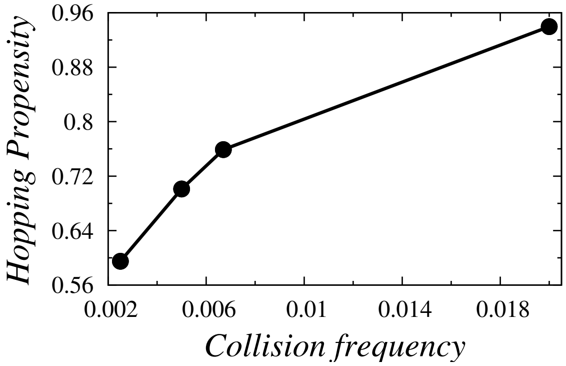

Figure S11: Correlation between crowder-DNA collisions and hopping propensity. To this end, we designed an artificial setup, where the system contains only a protein nonspecifically diffusing along the DNA contour. A random kick at a regular interval was applied on the searching protein mimicking the resultant of collisions on the protein imparted by the surrounding crowder molecules. The result clearly suggests that with increasing frequency of such random pushes, the sliding dynamics of the protein is hindered and the complementary hopping dynamics is promoted.

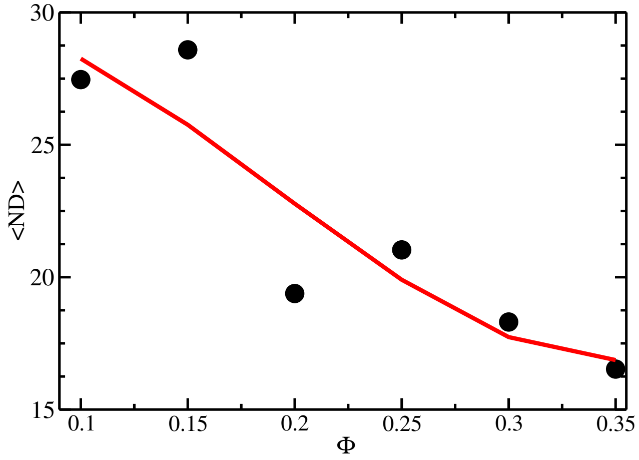

Figure S12: Variation of number of 3D diffusion events as a function of ϕ. As the concentration of crowder increases, the escape probability of the protein and thus the number of diffusion events decreases further. The crowders act as a barrier to hinder 3D diffusion.
